# Cardiac autonomic function in elderly patients with and without atrial fibrillation

**DOI:** 10.1093/ehjopen/oeag056

**Published:** 2026-04-04

**Authors:** Peter Hämmerle, Johannes Schier, Konstantinos D Rizas, Vincent Schlageter, Emel Kaplan, Stefanie Aeschbacher, Marius Rast, Philipp Krisai, Michael Coslovsky, Tobias Reichlin, Julia B Bardoczi, Nicolas Rodondi, Andreas S Müller, Alain M Bernheim, Giorgio Moschovitis, Maria Luisa De Perna, David Conen, Christian Sticherling, Stefan Osswald, Axel Bauer, Felix Mahfoud, Michael Kühne, Christine S Zuern

**Affiliations:** Department of Cardiology and Cardiovascular Research Institute Basel, University Hospital Basel, University of Basel, Petersgraben 4, 4031 Basel, Switzerland; Department of Cardiology and Cardiovascular Research Institute Basel, University Hospital Basel, University of Basel, Petersgraben 4, 4031 Basel, Switzerland; Medizinische Klinik und Poliklinik I, Klinikum der Universität München, Ludwig-Maximilians-Universität München, Ziemensstrasse 5, 80336 Munich, Germany; German Center for Cardiovascular Research (DZHK), Partner Site Munich Heart Alliance, Lazarettstrasse 36, 80636 Munich, Germany; Department of Cardiology and Cardiovascular Research Institute Basel, University Hospital Basel, University of Basel, Petersgraben 4, 4031 Basel, Switzerland; Department of Cardiology and Cardiovascular Research Institute Basel, University Hospital Basel, University of Basel, Petersgraben 4, 4031 Basel, Switzerland; Department of Cardiology and Cardiovascular Research Institute Basel, University Hospital Basel, University of Basel, Petersgraben 4, 4031 Basel, Switzerland; Department of Cardiology and Cardiovascular Research Institute Basel, University Hospital Basel, University of Basel, Petersgraben 4, 4031 Basel, Switzerland; Department of Cardiology and Cardiovascular Research Institute Basel, University Hospital Basel, University of Basel, Petersgraben 4, 4031 Basel, Switzerland; Department of Clinical Research, University of Basel, Spitalstrasse 8/12, 4031 Basel, Switzerland; Department of Cardiology, Inselspital, Bern University Hospital, University of Bern, Freiburgstrasse 20, 3010 Bern, Switzerland; Institute of Primary Health Care (BIHAM), University Bern, Mittelstrasse 43, 3012 Bern, Switzerland; Department of General Internal Medicine, Inselspital, Bern University Hospital, University of Bern, Freiburgstrasse, 3010 Bern, Switzerland; Institute of Primary Health Care (BIHAM), University Bern, Mittelstrasse 43, 3012 Bern, Switzerland; Department of General Internal Medicine, Inselspital, Bern University Hospital, University of Bern, Freiburgstrasse, 3010 Bern, Switzerland; Department of Cardiology, Triemli Hospital Zürich, Birmensdorferstrasse 497, 8055 Zürich, Switzerland; Department of Cardiology, Triemli Hospital Zürich, Birmensdorferstrasse 497, 8055 Zürich, Switzerland; Division of Cardiology, Ente Ospedaliero Cantonale (EOC), Cardiocentro Ticino Institute, Regional Hospital of Lugano, Via Tesserete 48, 6900 Lugano, Switzerland; Division of Cardiology, Ente Ospedaliero Cantonale (EOC), Cardiocentro Ticino Institute, Regional Hospital of Lugano, Via Tesserete 48, 6900 Lugano, Switzerland; Population Health Research Institute, McMaster University, 237 Barton Street East, Hamilton, Ontario L8L 2X2, Canada; Department of Cardiology and Cardiovascular Research Institute Basel, University Hospital Basel, University of Basel, Petersgraben 4, 4031 Basel, Switzerland; Department of Cardiology and Cardiovascular Research Institute Basel, University Hospital Basel, University of Basel, Petersgraben 4, 4031 Basel, Switzerland; Department of Internal Medicine III, Medical University of Innsbruck, Anichstrasse 35, 6020 Innsbruck, Austria; Department of Cardiology and Cardiovascular Research Institute Basel, University Hospital Basel, University of Basel, Petersgraben 4, 4031 Basel, Switzerland; Department of Cardiology and Cardiovascular Research Institute Basel, University Hospital Basel, University of Basel, Petersgraben 4, 4031 Basel, Switzerland; Department of Cardiology and Cardiovascular Research Institute Basel, University Hospital Basel, University of Basel, Petersgraben 4, 4031 Basel, Switzerland

**Keywords:** Atrial fibrillation, Periodic repolarization dynamics, Cardiac autonomic function, Heart rate variability

## Abstract

**Aims:**

Cardiac autonomic dysfunction is associated with an adverse prognosis in patients with atrial fibrillation (AF). However, the association of AF itself with cardiac autonomic function (CAF) remains unclear. We aimed to investigate whether CAF, assessed by heart rate variability (HRV), differs across patients with and without AF.

**Methods and results:**

We enrolled patients from a prospective multicentre study (Swiss-AF) with a 5-min resting ECG recording in SR or AF without pacing. Cardiac autonomic function was quantified by periodic repolarization dynamics (PRD), a marker of sympathetic activity, and by conventional HRV parameters. We included 2289 patients, 807 (35%) SR patients, 932 (41%) AF patients with SR ECGs (AF-SR), and 550 (24%) AF patients with AF ECGs (AF-AF). Mean age was 74 vs. 70 vs. 75 years; 37%, 31%, and 24% were female. Median PRD was 4.8 deg (IQR 2.6–6.7) in the SR group, 5.1 deg (IQR 2.9–6.9) in the AF-SR group, and 7.0 deg (IQR 5.8–8.4) in the AF–AF group (*P* < 0.001). After full adjustment (SR group = reference group), the AF–AF group showed a stronger association with elevated PRD (β-coefficient 2.10, 95% CI 1.79–2.41, *P* < 0.001) than the AF–SR group (β-coefficient 0.36, 95% CI 0.08–0.64, *P* = 0.011). Most other HRV parameters indicated greater autonomic impairment in the AF–SR group compared to the SR group.

**Conclusion:**

Atrial fibrillation was associated with increased sympathetic activity, with the greatest impairment observed in patients during AF, independent of cardiovascular risk factors. Periodic repolarization dynamics may represent a useful marker for the assessment of CAF in AF patients.

## Graphical Abstract

**Graphical Abstract:**
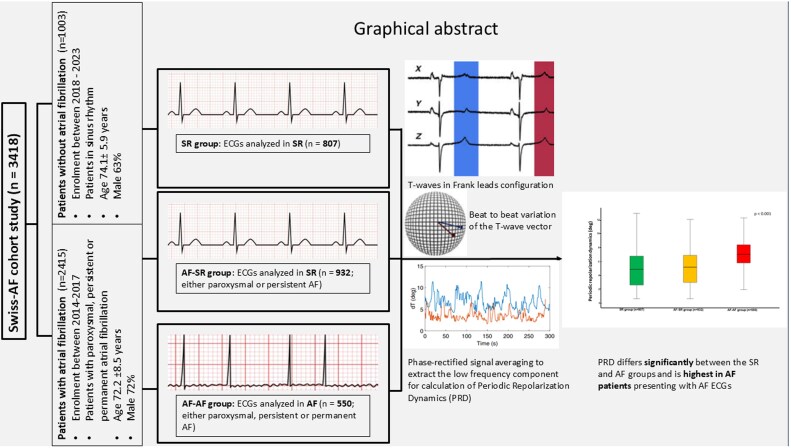

What’s New?

- This is the first study demonstrating that AF is associated with sympathetic overdrive, with the greatest autonomic imbalance observed in patients with presumably the highest AF burden.
- Contrary to traditional HRV parameters, PRD, a marker of sympathetic activity, overcomes disadvantages of classical HRV analyses, is independent of the presence of sinus rhythm, and provides valuable information regarding the cardiac autonomic nervous system, irrespective of cardiovascular risk factors.
- Periodic repolarization dynamics derived from short resting ECGs may provide a practical tool to assess cardiac autonomic dysfunction in AF. Future studies should determine its value for risk stratification and as a target for autonomic-modulating therapies.

## Introduction

Atrial fibrillation (AF) is a major global health burden, affects millions of individuals worldwide,^1^ and is linked to a broad range of adverse health events. The autonomic nervous system plays a key role in the onset, maintenance, and progression of AF. Heart rate variability (HRV) from ECG recordings is a standard measure of cardiac autonomic function (CAF).^2^ However, its application was mainly limited to patients in sinus rhythm (SR) so far.

Given the rich autonomic innervation of the atria,^3,4^ patients with AF may have a higher burden of autonomic dysfunction compared to SR patients, potentially contributing to their impaired prognosis.^5–8^ While risk stratification in AF patients by HRV may be clinically valuable, its measurement during AF remains unestablished.^2^ Only very few studies have reported on CAF and its prognostic value in AF populations.

Periodic repolarization dynamics (PRD) is a novel autonomic marker that reflects sympathetic that reflects sympathetic modulation of ventricular repolarization, quantified through analysis of T-wave dynamics.^9^ Unlike traditional HRV parameters, PRD does not rely on RR interval-based algorithms, making it a potentially more suitable tool for assessing CAF in AF patients, regardless of the current heart rhythm.

This study aims to evaluate CAF in AF patients and to investigate the association of AF with autonomic function by comparing CAF in patients with and without AF who share a comparable cardiovascular risk profile.

## Methods

### Patient population

This analysis is based on the Swiss Atrial Fibrillation (Swiss-AF) cohort (NCT02105844), an ongoing prospective study conducted at 14 study centres across Switzerland. Detailed information regarding study design has been published previously.^10^ In brief, the main inclusion criteria of Swiss-AF were previously documented AF and an age ≥65 years. Exclusion criteria were the inability to provide informed consent, any acute illness 4 weeks prior to enrolment, and short, reversible AF forms (e.g. septic patients). After enrolment of a total of 2415 AF patients between 2014 and 2017, the study was extended by recruiting another 1003 patients without AF from 2018 to 2023 (SR group). To this end, patients without a history of AF were screened and recruited at the respective clinics of each study centre. Patients were excluded if they had other rhythms than SR, contraindications to brain MRI, or dementia. Inclusion of patients with acute illness was delayed for 4 weeks. The study protocol has been approved by local ethic committees, and all participants gave informed consent. The study is in accordance with the Declaration of Helsinki. The data underlying this article will be shared on reasonable request to the corresponding author.

**Figure 1:**
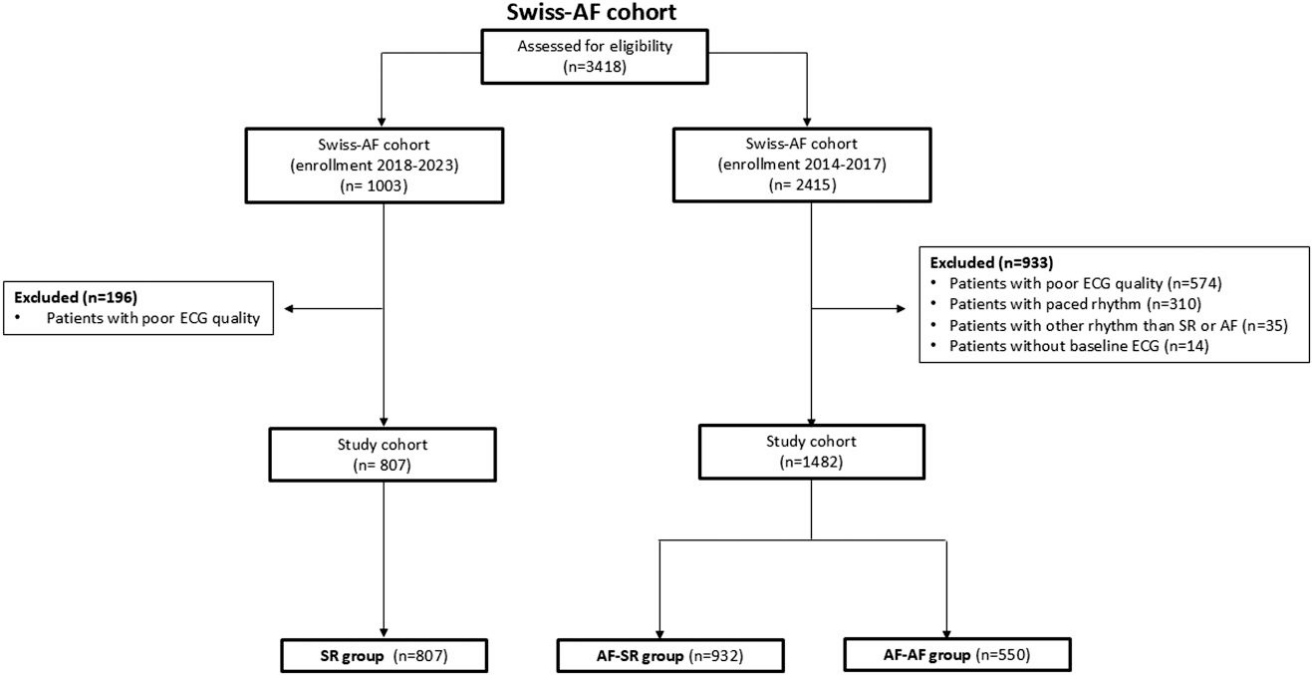
Flow diagram of patient selection.

For this analysis, 3418 patients were assessed for eligibility (***Figure 1***). A total of 933 patients from the AF–SR and AF–AF group of the Swiss-AF cohort were excluded for the following reasons: 574 patients due to low ECG quality, 310 with paced rhythm, 35 patients with other rhythm than SR or AF (e.g. atrial flutter), and 14 patients who had no ECG performed at baseline. From the SR group of the Swiss-AF cohort, we excluded 196 patients because of poor ECG quality. Consequently, 2289 patients were available for this analysis and were allocated into three groups according to their rhythm history and current rhythm on baseline ECG: (1) SR patients (*n* = 807; SR group), (2) AF patients in SR (*n* = 932; AF-SR group), and (3) AF patients in AF (*n* = 550; AF-AF group). Both the SR and AF–SR groups were in SR throughout the entire 5-min ECG recording. Patients in the SR group had no history of AF, whereas those in the AF–SR had a history of AF (e.g. paroxysmal or persistent AF).

### Clinical variables

Trained study personnel conducted an in-person study visit and gathered information about demographics, past medical history, medical and interventional treatment, and cardiovascular risk factors by using standardized case report forms. Blood pressure was estimated as the mean of three consecutive measurements. At the time of study entry, AF was categorized into paroxysmal, persistent, and permanent AF in accordance with contemporary guideline recommendations.^11^

### Assessment of cardiac autonomic function

All patients underwent a high-resolution 16-lead resting ECG recording for a total duration of 5 min under standardized resting conditions (standard 12-lead ECG plus C3R, C4R on the chest, and C7-C9 on the back). All recordings were stored digitally on a central server with a sampling frequency of 1 kHz (signal bandwidth 0.04–387 Hz) and a resolution of 1 µV/bit. All ECGs were visually inspected and manually annotated. ECGs that did not meet required quality criteria (e.g. loss of electrodes, noise, flawed T wave recognition, *n* = 770) were excluded. Periodic repolarization dynamics quantifies low-frequency (LF) oscillations (≤0.1 Hz) of cardiac repolarization, which reflect autonomic influences on the myocardium. Using high-resolution ECG in the orthogonal Frank lead configuration, each T-wave is represented as a vector (T°), and the angle between successive vectors (dT°) estimates instantaneous repolarization instability. The analysis of PRD has been described previously.^9^ For calculation of PRD, the raw ECG data were converted into a Frank leads configuration by inverse Dower transformation.^12^ T wave vectors were constructed for all T waves within the duration of ECG recording (e.g. 350 heartbeats within 5 min), reflecting deviations in space and time of each cardiac repolarization. To extract the LF component, phase-rectified signal averaging was applied^9^ (see Supplementary material online, *Figure S1*). Cardiac autonomic dysfunction was *a priori* defined according to established and previously published cut-offs of PRD (for the primary analysis, PRD ≥5.75 deg,^9^ and for exploratory analyses, PRD ≥7.50 deg^13^ and PRD ≥10.0 deg^14^). The following other HRV parameters were calculated in SR ECGs according to previously published algorithms^2,15^: deceleration capacity (DC) of heart rate, heart rate variability triangular index (HRVI), standard deviation of the normal-to-normal intervals (SDNN), root mean square of successive differences (rMSSD), power in the low-frequency (LF; 0.04–0.15 Hz) and high-frequency range (HF; 0.15–0.4 Hz). Frequency domain HRV measures were log-transformed. All autonomic parameters were calculated using a customized, open-source software (SMARTlab 1.5).^16^

### Statistical analysis

We summarized baseline characteristics stratified by the three rhythm groups (SR, AF–SR, AF–AF). Continuous variables were presented as means (standard deviations) or median (first and third quartile), if strongly skewed, and categorical variables were shown as frequencies (percentages). We compared HRV variables between the groups using Kruskal–Wallis test.

Linear regression models were used to examine the association between rhythm status and PRD, treated as a continuous outcome. Rhythm status was categorized into three groups: sinus rhythm without AF (SR), AF with sinus rhythm at the time of recording (AF–SR), and AF during AF (AF–AF). The SR group served as the reference category. To test difference in PRD between the AF–SR and AF–AF group, the Tukey post hoc comparison was used. *P* for linear trend across the groups was also calculated.

Binary logistic regression analysis was performed to explore the association between the three rhythm groups and cardiac autonomic dysfunction, defined by three different cut-offs for PRD (≥5.75 deg, ≥7.50 deg, and ≥10.0 deg). All regression analyses were performed unadjusted, age–sex adjusted, and multivariable adjusted for the following predefined variables: body mass index, actively or formerly smoking, history of hypertension, history of diabetes, history of heart failure, history of stroke, history of major bleeding, and comedication with beta-blockers and/or class Ic or III antiarrhythmics. Missing covariate measurements were very rare and were consequently imputed using mean values of continuous variables and the most frequent value for categorical variables. Results are displayed as beta-coefficients (β) or odds ratios (OR), according to model type, with the corresponding 95% confidence interval (CI). All statistical analyses were performed using SPSS 28.0.

## Results

The baseline characteristics of the 807 patients in the SR group, 932 patients in the AF–SR group, and 550 patients in the AF–AF group are summarized in *Table 1*. The mean age was 74 years in the SR group, 70 years in AF–SR group, and 75 years in the AF–AF group. Women comprised 37%, 31%, and 24% of each group, respectively. The mean CHA_2_DS_2_-VASc score was 3.4, 3.0, and 3.8. The clinical AF type varied between the AF–SR and AF–AF groups: paroxysmal AF was present in 67% vs. 16%, persistent AF in 33% vs. 29%, and permanent AF 55% vs. 0%. Rhythm control interventions, including electrical cardioversion (ECV) (38% vs. 33%) or pulmonary vein isolation (PVI) (36% vs. 6%), had been more frequently performed in the AF–SR group than in the AF–AF group. Class Ic and III antiarrhythmic drugs were prescribed in 29% of the AF–SR group compared to 9% of AF–AF patients (29% vs. 9%). Most patients with a history of AF (85% in the AF–SR and 94% in the AF–AF group) were on oral anticoagulants, while anticoagulant use in the SR group due to other reasons than AF was low (8%).

**Table 1:** Baseline characteristics of patients stratified by rhythm

Characteristic	SR group (*n* = 807)	AF–SR group (*n* = 932)	AF–AF group (*n* = 550)	*P*-value (SR vs. AF–SR vs. AF–AF)	*P*-value (AF–SR vs. AF–AF)
Age, years	74.1 ± 5.9	70.4 ± 8.5	75.1 ± 7.7	<0.001	<0.001
Females, *n* (%)	302 (37)	286 (31)	131 (24)	<0.001	0.005
Body mass index, kg/m^2^	26.3 ± 4.2	27.2 ± 4.7	27.8 ± 4.8	<0.001	0.013
Systolic blood pressure, mmHg	137 ± 17	137 ± 18	132 ± 19	<0.001	<0.001
Diastolic blood pressure, mmHg	76 ± 10	77 ± 11	79 ± 13	0.002	0.049
History of hypertension, *n* (%)	527 (65)	607 (65)	404 (74)	0.002	<0.001
History of diabetes mellitus, *n* (%)	133 (17)	124 (13)	105 (19)	0.010	0.003
Active and former smokers, *n* (%)	447 (55)	523 (56)	297 (54)	0.731	0.429
History of electrocardioversion, *n* (%)	—	351 (38)	182 (33)	n.a.	0.077
History of pulmonary vein isolation, *n* (%)	—	333 (36)	31 (6)	n.a.	<0.001
History of myocardial infarction, *n* (%)	165 (20)	105 (11)	96 (18)	<0.001	<0.001
History of heart failure, *n* (%)	101 (13)	146 (16)	175 (32)	<0.001	<0.001
CHA_2_DS_2_-VASc score, points	3.4 ± 1.4	3.0 ± 1.7	3.8 ± 1.7	<0.001	0.002
History of major bleeding, *n* (%)	35 (4)	47 (5)	38 (7)	0.106	0.136
Paroxysmal atrial fibrillation, *n* (%)	—	626 (67)	87 (16)	n.a.	<0.001
Persistent atrial fibrillation, *n* (%)	—	306 (33)	160 (29)	n.a.	0.089
Permanent atrial fibrillation, *n* (%)	—	—	303 (55)	n.a	n.a.
Antiarrhythmic therapy^a^, *n* (%)	—	268 (29)	47 (9)	n.a.	<0.001
Beta-blockers, *n* (%)	239 (30)	613 (66)	418 (76)	<0.001	<0.001
Non-vitamin K oral anticoagulants, *n* (%)	58 (7)	562 (60)	224 (41)	<0.001	<0.001
Vitamin K oral antagonists, *n* (%)	10 (1)	236 (25)	293 (53)	<0.001	<0.001

Data are means ± SD or counts (percentages). AF = atrial fibrillation, CHA_2_DS_2_-VASc = congestive heart failure, hypertension, age ≥75 years (two points), diabetes, prior stroke or TIA. n.a. = not applicable. SR = sinus rhythm.

^a^Class Ic and III according to VW classification.

### Cardiac autonomic function

**Figure 2:**
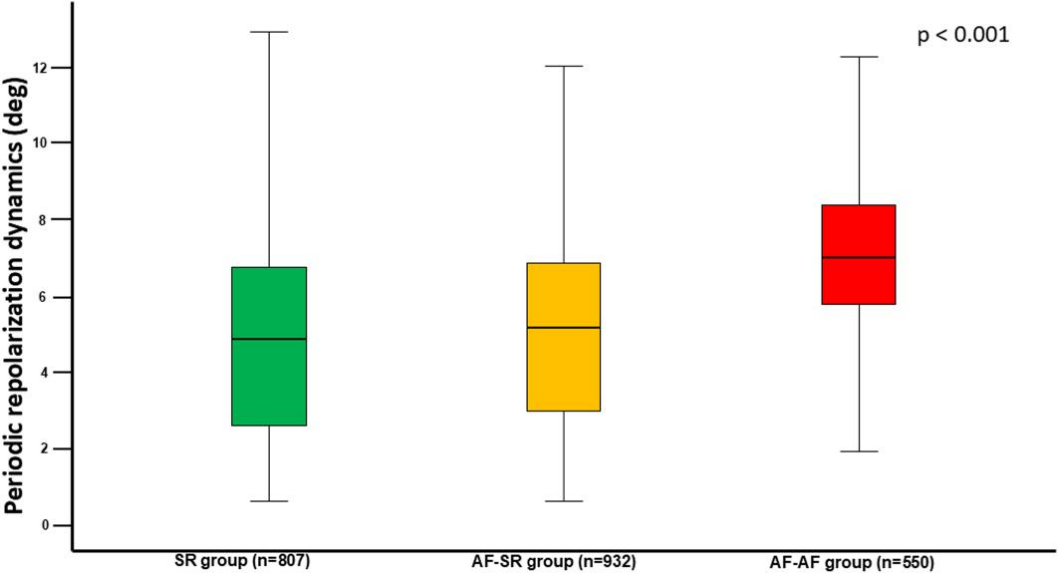
Periodic repolarization dynamics in sinus rhythm (SR) patients, atrial fibrillation patients presenting in sinus rhythm (AF-SR), and atrial fibrillation patients presenting in atrial fibrillation (AF-AF).

Heart rate variability parameters of the three rhythm groups are displayed in *Table 2*. Periodic repolarization dynamics values differed significantly across groups, with the lowest in SR patients and the highest in the AF–AF group (4.83 deg in SR vs. 5.13 deg in AF-SR vs. 6.98 deg in AF-AF, *P* < 0.001, *Figure 2*). Tukey *post hoc* comparisons revealed significant differences in PRD values between SR and AF–SR group (*P* = 0.035), SR and AF–AF group (*P* < 0.001), and AF–SR and AF–AF group (*P* < 0.001). Other parameters of HRV were more compromised in the AF–SR group than SR group, except for SDNN.

**Table 2:** Cardiac autonomic function stratified by rhythm

Autonomic parameter	SR group (*n* = 807)	AF–SR group (*n* = 932)	AF–AF group (*n* = 550)	*P*-value
PRD	4.83 (2.58–6.73)	5.13 (2.94–6.86)	6.98 (5.76–8.40)	<0.001
DC	4.64 (2.52–6.66)	3.78 (2.05–6.34)	n.a.	<0.001
HRVI	15.7 (13.1–19.1)	14.3 (11.7–18.0)	n.a.	<0.001
SDNN	90.1 (62.8–127)	86.0 (56.1–132)	n.a.	0.179
rMSSD	70.8 (48.0–106)	77.4 (48.0–122)	n.a.	0.025
Log LF	2.22 ± 0.58	2.13 ± 0.76	n.a.	0.020
Log HF	1.82 ± 0.57	1.83 ± 0.63	n.a.	0.684

Data are medians and interquartile ranges or means and standard deviations. *P*-value was obtained from Kruskal–Wallis test. Frequency domain measures of HRV have been log transformed. DC = deceleration capacity of heart rate. HF = high frequency (0.15–0.4 Hz). HRVI = heart rate variability triangular index. LF = low frequency (0.04–0.15 Hz). PRD = periodic repolarization dynamics. rMSSD = root mean square of successive differences. SDNN = standard deviation of the normal-to-normal interval

Cardiac autonomic dysfunction, defined as PRD ≥5.75 deg, was observed in 304 (38%) patients in the SR group, 397 (43%) the AF–SR group, and 416 (76%) the AF–AF group (*P* < 0.001). Additional PRD cut-offs and their corresponding prevalence rates are presented in the Supplementary material online, *Table S1* and Supplementary material online, *Figure S2*.

In the AF–SR group (*n* = 932), 36% of patients had undergone prior PVI before study inclusion. These patients showed significantly reduced standard HRV parameters compared with those without PVI, suggesting impaired CAF. Similarly, 38% had prior ECV, with modest reductions in some autonomic markers (DC, HRVI, SDNN, log LF), though differences were less pronounced than for PVI.

#### Association of atrial fibrillation with cardiac autonomic function

In linear regression analyses, PRD differed significantly across rhythm groups in univariable, age- and sex-adjusted, and multivariable-adjusted models (*Table 3*). In univariable linear regression analyses, PRD was higher in both AF groups compared with the SR reference group. Specifically, PRD was modestly increased in patients with AF who were in SR at the time of assessment (AF–SR; β 0.28, 95% CI 0.30–0.52; *P* = 0.028) and markedly increased in patients with AF who were in AF during the recording (AF–AF; β 2.20, 95% CI 1.92–2.48; *P* < 0.001). After adjustment for age and sex, these associations remained consistent, with a moderate increase in PRD in the AF–SR group (β 0.36, 95% CI 0.11–0.61; *P* = 0.005) and a pronounced increase in the AF–AF group (β 2.19, 95% CI 1.91–2.48; *P* < 0.001). In the fully adjusted multivariable model, PRD remained significantly higher in both AF groups compared with the SR group. The increase was moderate in the AF–SR group (β 0.36, 95% CI 0.08–0.64; *P* = 0.011) and substantially greater in the AF–AF group (β 2.10, 95% CI 1.79–2.41; *P* < 0.001). Post hoc comparisons confirmed that PRD was significantly higher in the AF–AF group than in the AF–SR group. A significant linear trend in PRD across rhythm groups was observed (see Supplementary material online, *Table S2*, linear regression model univariable analysis: β-coefficient 1.03; 95% CI: 0.89–1.18, *P* for linear trend <0.001, adjusted for age and sex: β-coefficient 1.03; 95% CI: 0.89–1.18, *P* for linear trend <0.001). This association remained significant after adjustment for multiple confounders across the three rhythm groups (β-coefficient 1.03; 95% CI: 0.87–1.19, *P* for linear trend <0.001).

**Table 3:** Linear regression model for association of the three rhythm groups (predictor) with periodic repolarization dynamics (outcome)

PRD (continuous)	Univariable model β (95% CI)	*P*-value	Age–sex-adjusted model β (95% CI)	*P*-value	Multivariable model β (95% CI)	*P*-value
AF–SR group	0.28 (0.30–0.52)	0.028	0.36 (0.11–0.61)	0.005	0.36 (0.08–0.64)	0.011
AF–AF group	2.20 (1.92–2.48)	<0.001	2.19 (1.91–2.48)	<0.001	2.10 (1.79–2.41)	<0.001

Data are beta-coefficients (β) [95% confidence intervals (CI)]. *P*-values were based on linear regression models. Sinus rhythm group was used as the reference group. AF = atrial fibrillation, PRD = periodic repolarization dynamics, Ref. = reference group, SR = sinus rhythm. Multivariable model was adjusted for age, sex, body mass index, actively or formerly smoking, history of hypertension, history of diabetes, history of heart failure, history of stroke, history of major bleeding, comedication with beta-blockers and/or Class Ic or III antiarrhythmics

In univariable logistic regression analyses, both AF groups were more likely to exhibit cardiac autonomic dysfunction, defined as PRD ≥5.75 deg, compared with the SR reference group. The odds of autonomic dysfunction were modestly increased in patients with AF who were in SR at the time of assessment (AF–SR; OR 1.23, 95% CI 1.01–1.49; *P* = 0.037) and markedly increased in patients with AF who were in AF during the recording (AF–AF; OR 5.14, 95% CI 4.04–6.54; *P* < 0.001).

These associations remained consistent after adjustment for age and sex, with higher odds of cardiac autonomic dysfunction in both the AF–SR group (OR 1.27, 95% CI 1.04–1.55; *P* = 0.017) and the AF–AF group (OR 5.16, 95% CI 4.05–6.58; *P* < 0.001).

In the fully adjusted multivariable model, AF remained independently associated with cardiac autonomic dysfunction. Compared with the SR group, the odds of PRD ≥5.75° were increased by 32% in the AF–SR group (OR 1.32, 95% CI 1.06–1.65; *P* = 0.014) and were more than fivefold higher in the AF–AF group (OR 5.05, 95% CI 3.86–6.60; *P* < 0.001, Supplementary material online, *Table S3*).

## Discussion

To the best of our knowledge, this is the first analysis to systematically investigate CAF in a population-based cohort of elderly patients with and without AF. Several important findings emerge from this study. First, AF was associated with an increased sympathetic activity, as reflected by levels of PRD, which progressively increased across the three rhythm groups despite similar baseline characteristics and comorbidities. Periodic repolarization dynamics was lowest in SR patients, higher in AF patients in SR (AF–SR group), and highest in AF patients with AF on ECG (AF–AF group, presumably those with greatest AF burden). This association remained significant after multivariable adjustment. Second, the prevalence of cardiac autonomic dysfunction was highest in the AF–AF group, regardless of PRD cut-off used. The association between rhythm and cardiac autonomic dysfunction was independent from covariables.

Prior studies of our group have demonstrated that impaired CAF, as assessed by HRV measures, is associated with all-cause mortality,^7^ silent brain infarcts,^8^ cognitive impairment,^6^ and stroke or systemic embolism^5^ in AF patients. However, these analyses were predominantly limited to analyses of ECGs in SR, missing out a large group of AF patients. Importantly, we were able to demonstrate that most traditional HRV parameters were reduced in the AF–SR group in comparison with the SR group. This finding is relevant, as conventional parameters were originally designed to assess CAF in SR patients.

There is a large body of evidence suggesting a strong link between the autonomic nervous system and AF. Paroxysmal AF can be classified as vagally mediated, adrenergically mediated, or mixed, depending on the predominant autonomic influence.^17^ Patients with vagal AF are typically younger men without significant cardiovascular disease, whereas adrenergic-mediated AF is often associated with significant structural heart disease and cardiovascular risk factors^17–19^ and is likely the dominant type in this elderly cohort with high rates of cardiovascular comorbidities. Elevated PRD in AF patients with AF on baseline ECG supports this hypothesis, as PRD reflects heightened sympathetic tone.

Before the onset of paroxysmal AF, an abnormal activation of the sympathetic and parasympathetic nervous system can be frequently observed.^20–22^ Of note, autonomic alterations can resolve after conversion to SR.^21^ Cardiac sympathetic activity increases during progression from paroxysmal to persistent AF,^22–24^ and atrial remodelling with increased sympathetic nerve densities has been found in patients with permanent AF.^25^ Our findings confirm that sympathetic activity is higher in patients with a presumably higher AF burden, e.g. the AF patients that have presented with AF ECGs, compared to AF patients that were in SR, after extensive multivariable correction of possible confounders.

Despite the well-established relationship between the autonomic nervous system and AF, quantifying the specific effect of AF on the autonomic nervous system has been challenging. This limitation arises from irregular RR intervals, which make conventional HRV analyses using conventional frequency and time domain measures infeasible.^2^ However, PRD, a novel autonomic marker, offers a viable solution by enabling the assessment of CAF in AF ECGs. This capability was already demonstrated in a recent *post hoc* analysis of the DANISH trial, which included approximately 20% of ECGs recorded during AF and where no significant interaction between PRD and AF was present for identifying patients, who benefit from prophylactic ICD implantation.^14^

Periodic repolarization dynamics focuses on the T wave and is not reliant on RR intervals. This parameter quantifies sympathetic activity of the myocardium which is organized in LF bursts of cardiac repolarization.^9^ This independence makes PRD particularly valuable for assessing CAF in AF patients, where traditional HRV measures fail. Various studies have demonstrated that a high PRD is a strong risk predictor of mortality in post-infarction patients.^9,26–28^ Moreover, PRD has been shown to predict mortality reductions associated with ICD implantation not only in non-ischaemic but also in ischaemic cardiomyopathy.^13,14^ It might be possible that AF patients with a high PRD and autonomic dysfunction will suffer from future adverse cardiovascular events. However, this hypothesis has not been tested yet. Of note, we found only weak correlations between PRD and standard HRV, suggesting that PRD provides information that is largely independent of HRV metrics.

Significant differences in PRD and most conventional HRV values between SR patients and AF patients in SR suggest that autonomic disturbances may persist in AF patients even during periods of SR. This supports the hypothesis that autonomic dysfunction is an intrinsic feature of AF, rather than merely a consequence of arrhythmia episodes. Identifying high-risk AF patients with autonomic dysfunction could allow for earlier individualized interventions, irrespective of rhythm status. Notably, in the AF–SR group, patients with prior PVI showed reduced values of standard HRV indices compared with those without PVI. Prior ECV was also associated with reduction of some HRV parameters. This may reflect either procedure-related autonomic effects or confounding by indication in patients with more advanced disease. However, we cannot provide the precise time interval between prior PVI or ECV and ECG acquisition to further explore this aspect in more detail.

Existing therapies targeting the autonomic nervous system in patients with AF, such as ablation of ganglionated plexi located in the epicardial fad pads next to the atria and pulmonary veins, on top of conventional PVI, may reduce the risk of AF recurrence.^18,19,29^ Renal denervation is another investigational strategy aimed at modulating autonomic tone to suppress extrapulmonary vein triggers for AF recurrence (Clinicaltrials.gov: NCT05817318). These approaches highlight the potential of autonomic modulation as a therapeutic avenue for AF management.

The observed association between AF, AF burden, and elevated sympathetic activity could have important clinical implications. Persistent autonomic dysfunction in AF patients, even during SR, may identify a high-risk subgroup prone to adverse cardiovascular events. Periodic repolarization dynamics could serve as a novel tool for risk stratification in AF cohorts, allowing earlier identification of patients who might benefit from intensified monitoring or targeted autonomic modulation therapies. Ultimately, incorporating autonomic assessment into routine AF management may improve individualized treatment strategies and reduce AF-related morbidity.

### Strengths and limitations

This study is strengthened by its large, well-characterized cohort and availability of more than 3400 digital high-resolution 16-lead ECGs. However, several limitations must be considered. First, HRV measures were derived from 5-min recordings, which may not fully capture long-term autonomic function. Second, the observational nature and cross-sectional design of this study preclude causal inference regarding the relationship between AF and cardiac autonomic dysfunction. Third, this was a secondary explorative analysis of the Swiss-AF cohort study, and generalizability to other populations is not applicable. Fourth, there was an overlap of PRD values among the three groups which reflects the known interindividual variability of PRD. However, statistically significant differences in PRD were observed between the groups, indicating a systematic shift in repolarization dynamics. Finally, although we adjusted for confounders in multivariable analyses, residual confounding by unmeasured clinical factors cannot be fully excluded.

## Conclusions

In this cross-sectional analysis of an elderly cohort with and without AF, AF was independently associated with an increased sympathetic tone and presence of cardiac autonomic dysfunction. Importantly, AF patients presenting with AF ECGs demonstrated the most pronounced autonomic disturbances. Despite the limitations associated with the observational, cross-sectional design of our study, these findings suggest that AF and AF burden significantly affect CAF. Whether AF patients with cardiac autonomic dysfunction have a worse prognosis than SR patients and whether patients with autonomic dysfunction would benefit from an intensified monitoring and therapy need to be determined in the future.

## Supplementary Material

oeag056_Supplementary_Data
