# Supplementary material for: Cardiac autonomic function in elderly patients with and without atrial fibrillation
Source: Eur Heart J Open. 2026 Apr 4;6(2):oeag056. doi: 10.1093/ehjopen/oeag056 (PMC13089404; doi:10.1093/ehjopen/oeag056)
Supplement: oeag056_Supplementary_Data [file oeag056_supplementary_data.zip › Supplement EHJ_Revision.docx]

**Supplement**

**Cardiac autonomic function in elderly patients with and without atrial fibrillation**

Peter Hämmerle MD^1^*, Johannes Schier MD^1^*, Konstantinos D. Rizas MD^2^, Vincent Schlageter PhD^1^, Emel Kaplan MD PhD^1^, Stefanie Aeschbacher PhD^1^, Marius Rast BSc^1^, Philipp Krisai MD^1^, Michael Coslovsky PhD^3^, Tobias Reichlin MD^4^, Julia B. Bardoczi MD^5,6^, Nicolas Rodondi MD^5,6^, Andreas S. Müller MD^7^, Alain M. Bernheim MD^7^, Giorgio Moschovitis MD^8^, Maria Luisa De Perna MD^8^, David Conen MD^9^, Christian Sticherling MD^1^, Stefan Osswald MD^1^, Axel Bauer MD^10^, Felix Mahfoud MD^1^, Michael Kühne MD^1^, Christine S. Zuern MD^1^, *on behalf of the Swiss-AF Investigators*

** Shared first authors*

**Abbreviations**

AF atrial fibrillation

CAF cardiac autonomic function

DC deceleration capacity of heart rate

HF power in the high frequency range (0.15–0.4 Hz)

HRVI heart rate variability triangular index

LF power in the low frequency range (0.04–0.15 Hz)

PRD periodic repolarization dynamics

rMSSD root mean square of successive differences

SDNN standard deviation of the normal-to-normal intervals

SR sinus rhythm

**Supplemental Table 1** Binary logistic regression model for association of the three rhythm groups (predictor) with cardiac autonomic dysfunction (outcome, defined by three established Cut-offs)

| **Cardiac autonomic dysfunction** | **Univariable Model**  **OR (95% CI)** | **p-value** | **Age-sex adjusted Model**  **OR (95% CI)** | **p-value** | **Multivariable Model**  **OR (95% CI)** | **p-value** |
| --- | --- | --- | --- | --- | --- | --- |
| **PRD ≥ 5.75 deg** | 2.11 (1.88 – 2.37) | <0.001 | 2.12 (1.89 – 2.38) | <0.001 | 2.18 (1.92 – 2.49) | <0.001 |
| **PRD ≥ 7.5 deg** | 1.96 (1.71 – 2.24) | <0.001 | 1.93 (1.68 – 2.21) | <0.001 | 1.92 (1.66 – 2.22) | <0.001 |
| **PRD ≥ 10.0 deg** | 2.26 (1.72 – 2.96) | <0.001 | 2.15 (1.64 – 2.82) | <0.001 | 2.11 (1.58 – 2.83) | <0.001 |

Data are odds ratios (OR) (95% confidence intervals [CI]). P-values were based on logistic regression models. PRD = periodic repolarization dynamics. Cardiac autonomic dysfunction was defined as PRD≥5.75, PRD ≥ 7.5 deg and PRD ≥ 10.0 deg. Multivariable model was adjusted for age, sex, body mass index, active or former smoking, history of hypertension, history of diabetes, history of heart failure, history of stroke, history of major bleeding, comedication with beta-blockers and/or class Ic or III antiarrhythmics

**Supplemental Table 2** Linear regression model for association of the three rhythm groups (predictor) with periodic repolarization dynamics (outcome)

| **PRD (continuous)** | **Univariable Model β (95% CI)** | **p for linear trend** | **Age-sex adjusted Model**  **β (95% CI)** | **p for linear trend** | **Multivariable Model**  **β (95% CI)** | **p for linear trend** |
| --- | --- | --- | --- | --- | --- | --- |
| **Three rhythm groups (SR, AF-SR, AF-AF)** | 1.03 (0.89 – 1.18) | <0.001 | 1.03 (0.89 – 1.18) | <0.001 | 1.03 (0.87 – 1.19) | <0.001 |

Data are beta-coefficients (β) (95% confidence intervals [CI]). P-values were based on linear regression models. PRD = periodic repolarization dynamics, used as a continuous variable. Multivariable model was adjusted for age, sex, body mass index, active or former smoking, history of hypertension, history of diabetes, history of heart failure, history of stroke, history of major bleeding, comedication with beta-blockers and/or class Ic or III antiarrhythmics

**Supplemental Table 3** Binary logistic regression model for association of the three rhythm groups (predictor) with cardiac autonomic dysfunction* (outcome).

| **Cardiac autonomic dysfunction (PRD≥5.75 deg)** | **Univariable Model**  **OR (95% CI)** | **p-value** | **Age-sex adjusted Model**  **OR (95% CI)** | **p-value** | **Multivariable Model**  **OR (95% CI)** | **p-value** |
| --- | --- | --- | --- | --- | --- | --- |
| **AF-SR** | 1.23 (1.01-1.49) | 0.037 | 1.27 (1.04-1.55) | 0.017 | 1.32 (1.06-1.65) | 0.014 |
| **AF-AF** | 5.14 (4.04-6.54) | <0.001 | 5.16 (4.05-6.58) | <0.001 | 5.05 (3.86-6.60) | <0.001 |

Data are odds ratios (OR) (95% confidence intervals [CI]). P-values were based on logistic regression models. The SR group was used as the reference group. PRD = periodic repolarization dynamics. Multivariable model was adjusted for age, sex, body mass index, active or former smoking, history of hypertension, history of diabetes, history of heart failure, history of stroke, history of major bleeding, comedication with beta-blockers and/or class Ic or III antiarrhythmics

*cardiac autonomic dysfunction defined as PRD≥5.75 deg.
