# Supplementary figures and images for: Cardiac autonomic function in elderly patients with and without atrial fibrillation
Source: Eur Heart J Open. 2026 Apr 4;6(2):oeag056. doi: 10.1093/ehjopen/oeag056 (PMC13089404; doi:10.1093/ehjopen/oeag056)

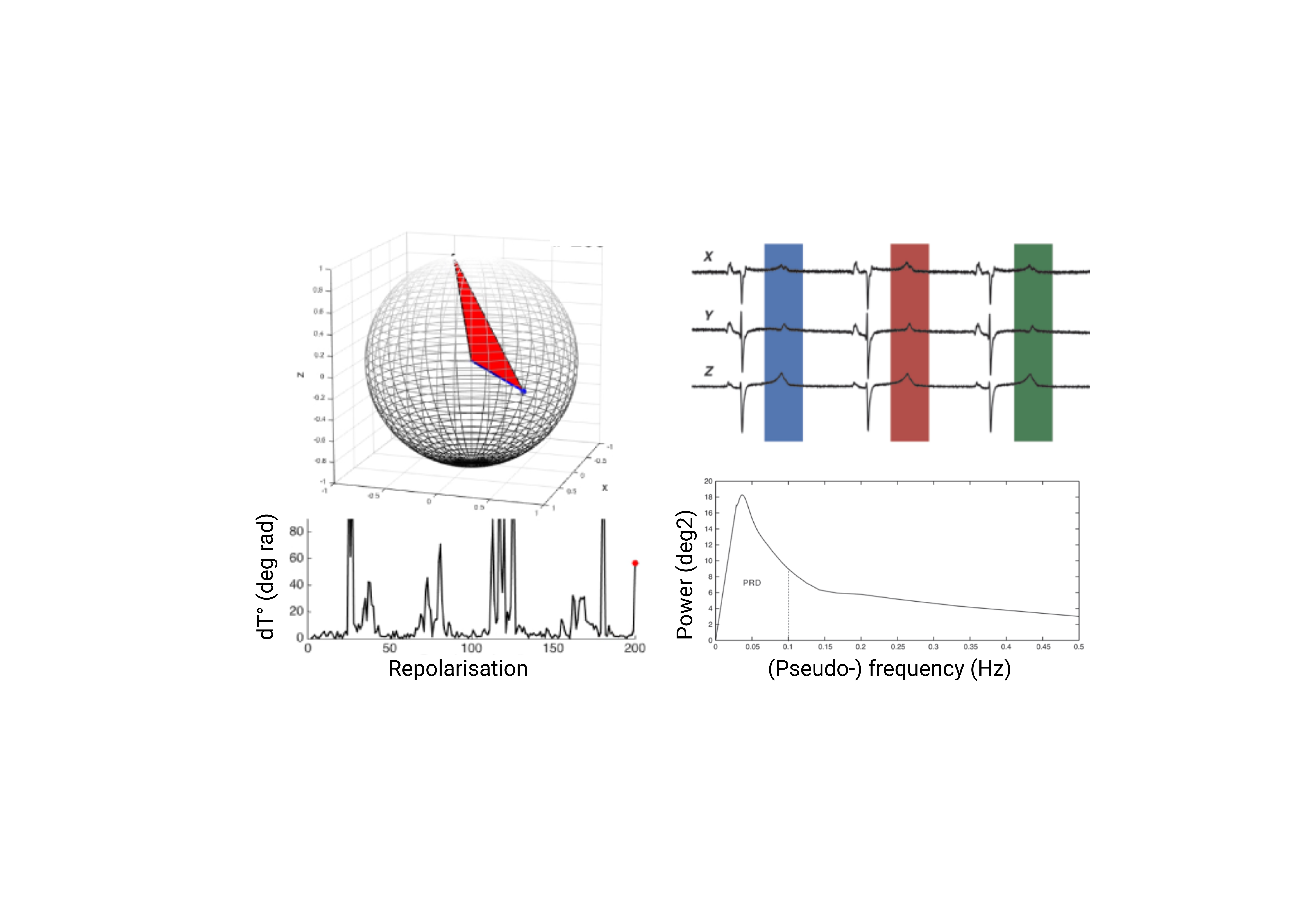

Supplement: oeag056_Supplementary_Data [file oeag056_supplementary_data.zip › Supplemental figure 1.png]

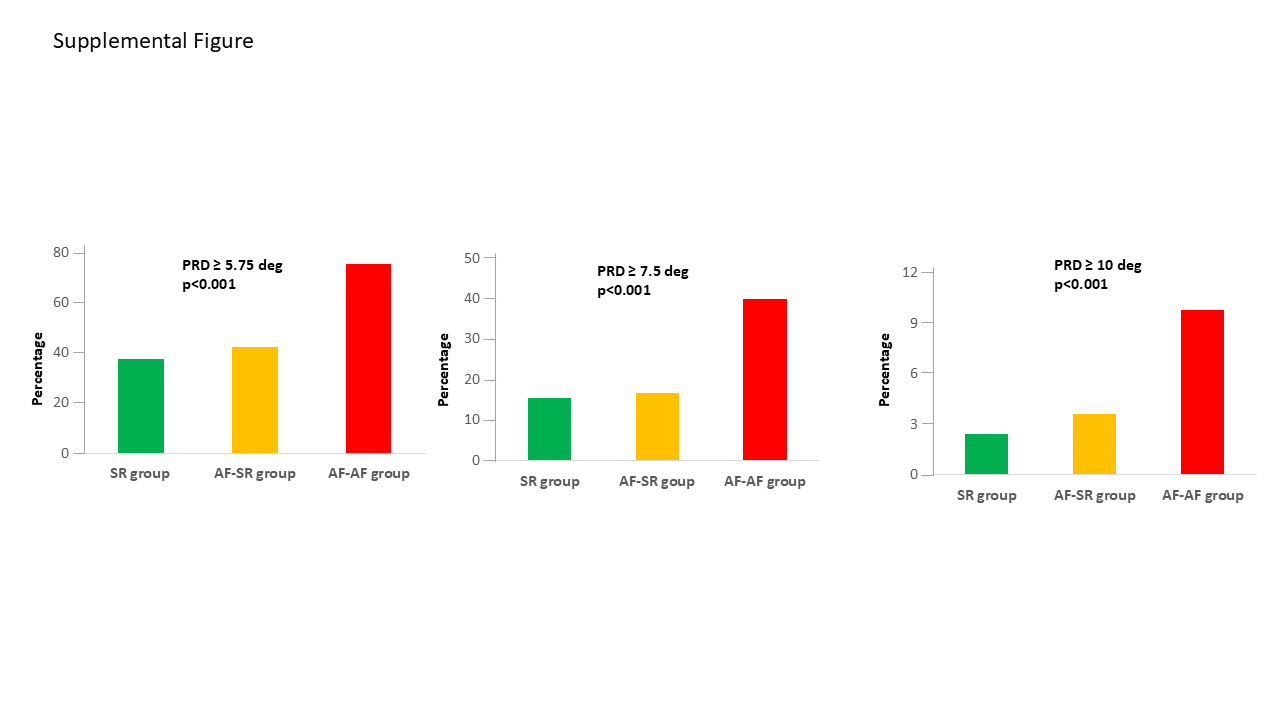

Supplement: oeag056_Supplementary_Data [file oeag056_supplementary_data.zip › Supplemental figure 2.tif]
